# Transient Receptor Potential Melastatin 8 (TRPM8)-Based Mechanisms Underlie Both the Cold Temperature-Induced Inflammatory Reactions and the Synergistic Effect of Cigarette Smoke in Human Bronchial Epithelial (16HBE) Cells

**DOI:** 10.3389/fphys.2019.00285

**Published:** 2019-03-29

**Authors:** Jing Wang, Gang Yang, Minchao Li, Xiangdong Zhou

**Affiliations:** ^1^Department of Respiratory Medicine, The Second Clinical Hospital of Chongqing Medical University, Chongqing, China; ^2^Department of Neurosurgery, The First Clinical Hospital of Chongqing Medical University, Chongqing, China; ^3^Department of Respiratory Medicine, The First Affiliated Hospital of Hainan Medical University, Haikou, China

**Keywords:** transient receptor potential melastatin 8, cold temperature, cigarette smoke extract, chronic obstructive pulmonary disease, airway inflammation

## Abstract

Transient receptor potential melastatin 8 (TRPM8) is a major receptor of cold environment. Recently, we found that cigarette smoke extract (CSE) upregulated TRPM8 mRNA and protein expression in bronchial tissues that made them more sensitive to cold stimuli. In our present study, we found that cold temperature (18°C)-induced activation of TRPM8 in 16HBE (human bronchial epithelial) cells facilitated Ca^2+^ influx and subsequently led to the increased expression of interleukin (IL)-6, IL-8, and tumor necrosis factor (TNF)-α via the upregulation of p-extracellular signal-regulated kinase (ERK) and the activation of NF-κB. In addition, 16HBE cells that co-stimulated with 18°C and CSE were used to explore the synergistic effect of CSE on cold temperature-induced inflammatory cytokine production as well as the possible involved signaling pathway. RT-PCR and western blot analysis revealed that CSE upregulated TRPM8 mRNA and protein level in 16HBE cells. Ca^2+^ imaging, western blot, and luciferase assay showed more robust increase in intracellular Ca^2+^ and promoted phosphorylated ERK, P38, and NF-κB activity, respectively, in 16HBE cells co-stimulated with CSE and cold temperature, and such alteration was attenuated by TRPM8 short hairpin RNA (shRNA) transfection and BCTC pretreatment. Furthermore, enhanced levels of IL-6, IL-8, and TNF-α showed by enzyme-linked immunosorbent assay (ELISA) were reduced by specific inhibitors of ERK and NF-κB. Collectively, our results suggest that mitogen-activated protein kinase (MAPK)/NF-κB signaling is involved in TRPM8-mediated cold temperature-induced inflammatory cytokine expression. In addition, CSE synergistically amplifies cold temperature-induced inflammatory factors release via upregulating TRPM8 expression and enhancing MAPK/NF-κB signaling pathway.

## Introduction

Exposure to cold temperature is a primary environmental factor for the exacerbation of chronic obstructive pulmonary disease (COPD) that results in decline in lung function and quality of life as well as an increased risk of mortality (Donaldson et al., 1999; Tseng et al., 2013; McCormack et al., 2017). A better understanding of the mechanism of cold temperature-provoked COPD exacerbation will help to prevent and manage exacerbations, thus improving these patients’ quality of life and reducing the burden of COPD.

The mechanisms of cold temperature’s action mainly depend on the cold-sensing transient receptor potential (TRP) channel TRP melastatin 8 (TRPM8), a ligand-gated, Ca^2+^-permeant, non-selective cation channel (McKemy et al., 2002; Yin et al., 2018). Its expression was initially discovered in some cold-sensitive primary afferent neurons and is activated by temperatures of less than 28°C (Bautista et al., 2007) and cooling agents, like menthol, eucalyptol, and icilin (Peier et al., 2002; Behrendt et al., 2004; Malkia et al., 2007; Voets et al., 2007). TRPM8 is also expressed in various non-neuronal tissues, including prostate tissue (Tsavaler et al., 2001; Abe et al., 2005; Zhang and Barritt, 2006), testicular tissues (Stein et al., 2004), and lung tissue (Sabnis et al., 2008; Li et al., 2011). Cold temperature or cooling agents-induced activation of TRPM8 in lung epithelial cells is found coupled with enhanced expression of substantial cytokine and chemokine, including interleukin (IL)-1α, IL-1β, IL-4, IL-6, IL-8, IL-13, tumor necrosis factor (TNF)-α, and granulocyte-macrophage colony-stimulating factor (GM-CSF), and these cold- or cold agent-induced effects can be blocked with treatment with TRPM8 antagonists or TRPM8 short hairpin RNA (shRNA) (Ashwini et al., 2008; Liu et al., 2018), suggesting the role of epithelial TRPM8 in airway inflammation. However, the involved signaling pathway is not completely understood.

Nuclear factor-κB (NF-κB) signaling and mitogen-activated protein kinase (MAPK) family members, such as extracellular signal-regulated kinase (ERK), c-Jun-N-terminal kinase (JNK), and p38, have been explored as upstream signaling intermediates responsible for the production of inflammatory cytokines(Sun et al., 2013; Wang et al., 2017). Recent studies revealed that TRPM8 can upregulate the phosphorylation of MAPK family and increase the activity of NF-κB (Lin et al., 2017). Therefore, we speculated that MAPK/NF-κB signal pathway participated in TRPM8-mediated inflammatory cytokine release in airway epithelium.

Our previous results have demonstrated that TRPM8 located in the bronchial epithelium was upregulated in subjects with COPD, and subsequent research has confirmed that cigarette smoke inhalation enhanced the expression of TRPM8 in bronchial tissue. Cigarette smoke has been considered a main etiologic factor in the pathogenesis of COPD. Given the cold-activation characteristics of TRPM8 and the fact that its mRNA and protein can be upregulated by cigarette smoke, we postulated that the concomitant presence of cigarette smoke would result in a TRPM8-mediated synergistic enhancement of airway inflammation induced by cold temperature.

To test our hypotheses, we exposed 16HBE (human bronchial epithelial) cells to cold temperature (18°C) and/or cigarette smoke extract (CSE), and the responses of TRPM8 expression, intracellular Ca^2+^, MAPK/NF-κB signaling, and inflammatory cytokines, including IL-6, IL-8, and TNF-α, to these stimuli were compared. TRPM8 shRNA transfection and TRPM8 antagonist BCTC were used to verify the role of TRPM8 in cold temperature-induced inflammatory reactions and the synergistic effect of cigarette smoke.

## Materials and Methods

### Reagents

The human bronchial cell line 16HBE was purchased from American Type Culture Collection (ATCC, Rockville, MD, United States). Cell culture medium (RPMI 1640) and fetal bovine serum (FBS) were obtained from HyClone (Logan, UT, United States). 3.3-(4,5-Dimethylthiazol-2-yl)-2, 5-diphenyltetrazolium (MTT) was purchased from Beyotime Biotechnology (Beijing, China). Rabbit polyclonal antibodies against phospho-ERK, ERK, p-p38, and p38 were obtained from Santa Cruz Biotechnology, Inc. (Santa Cruz, CA, United States). Rabbit polyclonal antibodies against phospho-JNK, JNK were obtained from Cell Signaling Technology (Beverly, MA, United States). Rabbit polyclonal antibody against TRPM8 was purchased from Abcam (Cambridge, MA, United States). Mouse monoclonal antibody against IκBα was from Cell Signaling Technology (Danvers, MA, United States). Fluo3 acetoxymethylester (Fluo3-AM), the double luciferase reporter gene assay kit, and p-NF-κB Luc luciferase reporter vector were purchased from ByoTime Technology (Beijing, China). pRL-TK Renilla luciferase reporter vector was obtained from Promega (Madison, WI, United States). The PrimeScript RT kit and SYBR Premix Ex Taq TM were obtained from TaKaRa Biotechnology (Dalian, China). Exon 18-specific TRPM8 shRNA and scramble shRNA were purchased from Santa Cruz Biotechnology. BCTC was a kind gift from Prof. Christopher A. Reilly (University of Utah, Salt Lake City). Effectene transfection reagent was obtained from Qiagen, (Hilden, Germany).G418 were purchased from Invitrogen (San Diego, CA, United States). All the primers were synthesized by Sangon Biotech Co., Ltd. (Shanghai, China). IL-6, IL-8, and TNF-α enzyme-linked immunosorbent assay (ELISA) kits were obtained from Jingmei Biotech Co., Ltd. (Shenzhen, China). Inhibitors of ERK (U0126), p38 (SB203580), and NF-κB (BAY 11-7082) were purchased from ByoTime Technology (Beijing, China).

### Preparation of CSE

The CSE was prepared as previously described (Guo et al., 2016). Briefly, each commercial cigarette was obtained from the Chongqing Cigarette Factory (Chongqing, China) and was smoked for 5 min with a 60-ml syringe through 10 ml of serum-free medium. The generated extract was sterile-filtered with a 0.22-μm filter (Millipore, Bedford, MA, United States), adjusted to pH 7.4, and then stored at −80°C. This cigarette smoke concentration was defined as 100% and was further diluted with serum-free medium to the required concentration (3%).

### Cell Culture and Grouping

The 16HBE cells were grown in RPMI 1640 medium containing FBS (10%), penicillin G (50 IU/ml), streptomycin (100 mg/l), and HEPES buffer (25 mmol/l) and cultured in six-well plates at 37°C in an air-ventilated, humidified incubator maintained with 5% carbon dioxide. The medium was replaced with fresh medium routinely; when the cells reached 60–80% confluence, a passage operation was performed.

Our first part of experiment was designed to determine whether MAPK/NF-κB signaling participates in TRPM8-mediated, cold-induced inflammatory cytokine production in the respiratory epithelia. Cultured cells were divided into a control group and a group exposed to cold temperature; cells in the control group were maintained at a temperature of 37°C, whereas cells treated with cold temperature were exposed to 18°C in a temperature-controlled incubator for 4 h followed by a recovery of 2 h at 37°C, as previously described (Ashwini et al., 2008). Inhibition experiments were evaluated by transfected 16HBE cells with TRPM8 shRNA/scramble shRNA or by adding the TRPM8 antagonist BCTC (15 μmol/l) before the cold treatment. Furthermore, to study the possible role of the MAPK/NF-κB signaling, specific inhibitors of this signaling pathway were added [inhibitors of ERK (U0126), p38 (SB203580), and NF-κB (BAY 11-70) were added 2, 2, and 3 h, respectively, before cold exposure].

The second part of our experiment was designed to investigate the effects of cold temperature on CSE-induced airway inflammation via the TRPM8 channel. In this experiment, cultured cells were divided into a control group, a CSE exposure group, and a group exposed to both CSE and 18°C. Based on the fact that CSE increases the levels of airway inflammatory cytokines in a concentration-dependent manner and time-dependent manner (Wu et al., 2014; Lin et al., 2015), 3% CSE exposure for 24 h was chosen in our present experiment (Guo et al., 2016; Li et al., 2016). Cells in the group exposed to both CSE and 18°C were incubated in the presence of 3% CSE for 20 h before the exposure to 18°C for another 4 h. To investigate the possible roles of TRPM8 and MAPK/NF-κB signaling in the synergistic effect of cold temperature on CSE-induced airway inflammation, TRPM8 shRNA/scramble shRNA transfection, BCTC pretreatment, and specific inhibitors intervention of MAPK/NF-κB signaling pathway were performed as in the first experiment.

The supernatants and cells were collected after another 4 h of culturing for quantification of TRPM8, IL-6, IL-8, and TNF-α mRNA and protein expression.

### Cell Viability Assay

To exclude the possibility that cytokine gene induction was the consequence of changes in cell membrane integrity or overt injury to cells, cell viability was determined by MTT assay as previously described (Li et al., 2011). At 8, 16, 24, and 36 h, 20 μl of 5 mg/ml MTT was added to the designated wells, and cells were incubated at 37°C for another 4 h. The supernatants were then abandoned and 150 μl of dimethyl sulfoxide was added, and then the plate was placed on an orbital shaker at room temperature. After 15 min, absorbance was read at 570 nm using a microplate reader. The assay was carried out with four replicates for each group.

### Stable Transfection of 16HBEs With TRPM8shRNA and Scramble shRNA

According to the manufacturer’s instructions, 16HBE cells were transfected with exon 18-specific TRPM8 shRNA or scramble shRNA by Effectene transfection reagent (10:1 reagent-to-DNA). Stably transfected cells were screened by means of administration of G418 (300 μg/ml). Meanwhile, knockdown in TRPM8 expression was analyzed with western blotting.

### Ca^2+^ Imaging Analysis of Intracellular Calcium Concentration

As previously described (Li et al., 2011), cultured 16HBE cells were loaded with the membrane-permeable fluorogenic Ca^2+^ indicator Fluo-3-AM ester for 40 min at 37°C according to the manufacturer’s instructions. Cells were then washed three times with modified Krebs-Ringer HEPES buffer and then incubated at 37°C for an another 30 min before analysis. Images were collected at multiple time points after exposure to cold temperature (18°C) and/or CSE with a laser scanning confocal microscope system (Leica TCSSP2). Fluo-3 was excited by argon laser light at 480 nm and fluorescence was measured at wavelengths of 530 nm. As to the inhibition experiments, 16HBE cells were transfected with TRPM8 shRNA/scramble shRNA or pretreated with BCTC followed by exposure of 18°C and/or CSE. Finally, the fluorescence intensity was analyzed with the quantification tools in the confocal microscope software.

### Quantification of TRPM8, p-ERK, p-p38, p-JNK, and IκBα Protein Expression

Western blot was carried out and assessed as previously described (Yu and Li, 2017).

### Luciferase Assay for NF-κB Activity

As previously described (Li et al., 2011), 16HBE cells approaching 70–80% confluence were transfected with pNF-κB-luc plasmid and the control pRL-TK Renilla luciferase reporter plasmid using Lipofectamine. Approximately 12 h after transfection of cells were incubated according to the experimental protocol for additional 4 h. The cells were harvested and the activity of NF-κB was assayed according to the manufacturer’s instructions given in the luciferase reporter gene assay kit. The ultimate luciferase activity of each sample was expressed as the ratio of its detected luciferase relative light units (RLUs) to the Renilla luciferase activity.

### Quantification of IL-6, IL-8, and TNF-α Protein Expression by ELISA

The specific procedure was carried out based on the manufacturer’s instructions.

### Quantification of TRPM8, IL-6, IL-8, and TNF-α Gene Expression With Quantitative Real-Time PCR (qPCR)

The TRIzol reagent was used to extract total RNAs from the cultured16HBE cells in each group. Next, the extraction was confirmed by agarose gel electrophoresis with an absorbance (A260/280) value between1.8–2.0. Reverse transcription into complementary DNA was conducted with an RT-PCR kit. All PCR primers were from Sangon Biotech (Shanghai, China). The PCR primers for TRPM8, IL-6, IL-8, TNF-α, and β-actin were designed based on the published sequences (see Table 1). A 25-μl PCR system was prepared according to the kit’s instructions. The amplification was performed for 40 cycles under the following conditions: pre-denaturation at 95°C for 3 min; denaturation at 95°C for 15 s; annealing at 56°C (IL-6), 58°C (IL-8, TRPM8, and β-actin), for 1 min, and extension at 72°C for 10 min. The relative mRNA expression of the target gene was calculated with the 2^−ΔΔCt^ method, where Ct, the threshold cycle, is the cycle number at which emitted fluorescence reaches the threshold value. ΔCt = (Ct_*targetgene*_ −Ct_*house*−*keepinggene*_), and ΔΔCt is the difference between the control group and experimental group ΔCt values.

**Table 1:** Primer sequences used for RT-PCR analysis of selected cytokine genes.

Gene	Sense (5′-3′)	Antiense(3′-5′)	Product size (bp)	GenBank accession no.
TRPM8	CAGCGCTGGAGGTGGATATTC	CACACACAGTGGVTTGGACTC	144	NM024080
GAPDH	TGTTCGTCATGGGTGTGAACC	CATGAGTCCTTCCACGATACC	137	NM002046
IL-6	CTTCTCCACAAGCGCCTT	GGCAAGTCTCCTCATTGAATC	328	NM000600
IL-8	GTGGCTCTCTTGGCAGCCTTC	CAGGAATCTTGTATTGCATCT	410	NM000584
TNF-α	GAGTGACAAGCCTGTAGCCCATGTTGTAGC	GCAATGATCCCAAAGTAGACCTGCCCAGACT	444	AF0433442

### Statistical Analysis

SPSS 22.0 software program (IBM Corporation, Armonk, NY, United States) was used in this study. All the results are expressed as the mean ± SD, and statistical analysis was performed using one-way analysis of variance (ANOVA) for multiple group comparisons and Student’s *t*-test (two-tailed) for comparisons between two groups. A *p*-value of less than 0.05 was considered statistically significant.

## Results

### Cell Viability

**FIGURE 1:**
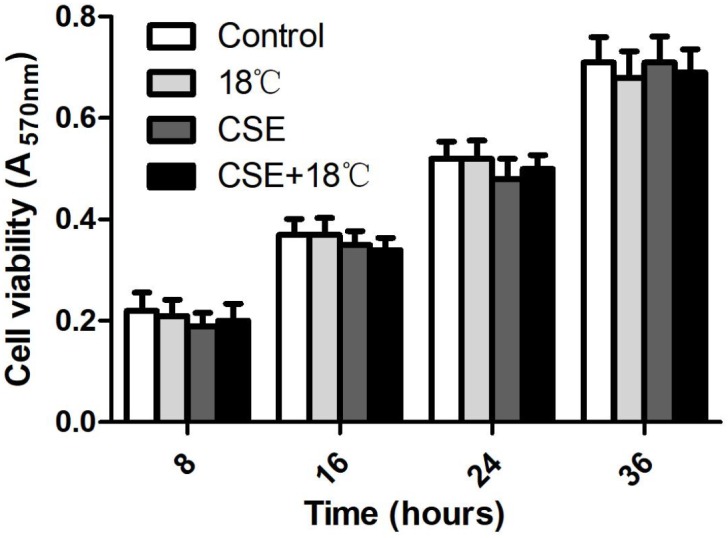
Cell viability unaltered by cold temperature (18°C) and/or CSE (3%) at 8, 16, 24, and 36 h that determined by MTT assay. Data are expressed as mean ± SD (*n* = 4), one-way ANOVA.

We used the MTT assay to test whether cold temperature (18°C) and/or CSE concentration (3%) exerted a cytotoxic effect on 16HBE cells. There was no significant difference in cell viability among different cell groups at 8, 16, 24, and 36 h (Figure 1, *p* > 0.67), indicating that exposure to 18°C and/or 3% CSE was essentially non-toxic to 16HBE cells under conditions that prompted Ca^2+^ influx, and the increased cytokine release was not ascribed to changes in cell permeability or obvious damage to cells. In addition, cell viability in groups with TRPM8 shRNA or scramble shRNA transfection, pretreatment with BCTC and specific inhibitors of ERK (U0126), p38 (SB203580), or NF-κB (BAY 11-708) did not show significantly difference compared with that in the control (data not shown).

### Effects of Cigarette Smoke and Cold Temperature on TRPM8 Expression

**FIGURE 2:**
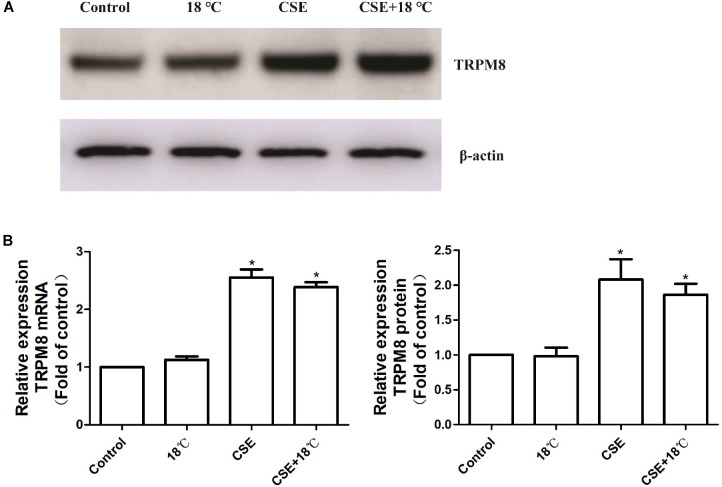
Effects of cold temperature or/and CSE on TRPM8 expression in 16HBE cells relative quantification of transient receptor potential melastatin 8 (TRPM8) mRNA and protein in 16HBE cells exposed to18°C or/and CSE. **(A)** The levels of the TRPM8 protein were determined by western blot analysis. **(B)** The levels of TRPM8 mRNA were determined by real-time RT-PCR, and a comparative Ct method (2^−ΔΔCt^) was used for the relative mRNA quantification. **(C)** Densitometry quantification of the bands in **A** was performed using Quantity One software, and the results are expressed as the ratio of the expression of TRPM8 to β-actin. The values in **B** and **C** are shown as the means ± SD; *n* = 4. ^∗^*p* < 0.05 vs. control.

Consistent with our previous animal study, PCR and western blot analysis showed that exposure of 16HBE cells to 3% CSE for 24 h amplified the mRNA (2.39 ± 0.17) and protein (2.08 ± 0.58) levels of TRPM8 (Figure 2), which in the control group were 1.0 ± 0.00 and 1.0 ± 0.00, respectively (both *p* < 0.01). By contrast, the exposure to 18°C did not alter either the mRNA or the protein level of TRPM8 (both *p* > 0.05 compared with control). Further analysis demonstrated that 16HBE cells exposed to both 18°C and CSE expressed similar levels of mRNA (2.55 ± 0.28) and protein (1.86 ± 0.31) of TRPM8 compared with those in the group exposed to CSE alone (*p* = 0.68, and *p* = 0.57, respectively).

### Effect of TRPM8 on the Increased Intracellular Calcium Concentration Induced by Cold Temperature and/or CSE

**FIGURE 3:**
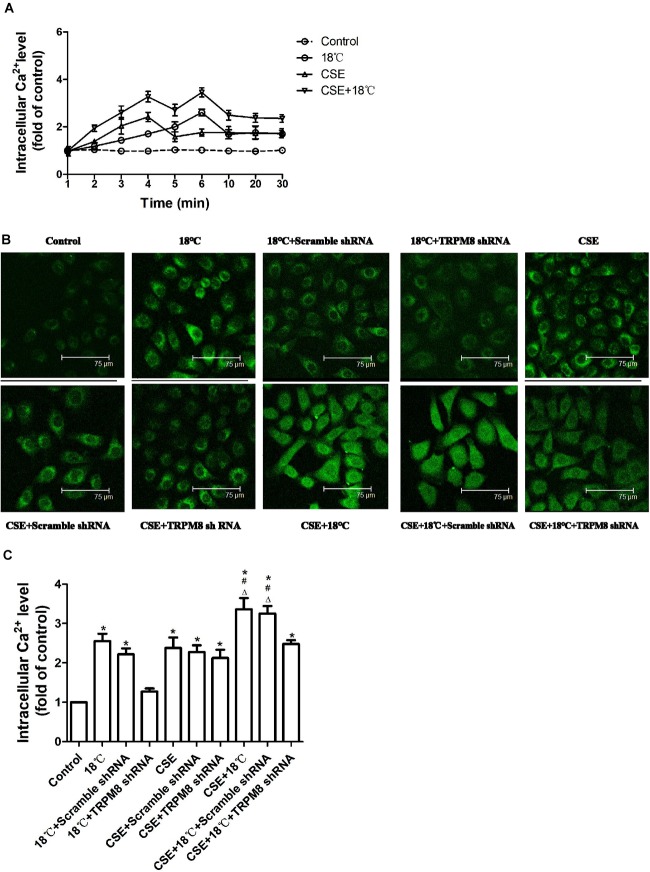
Roles of cold temperature or/and CSE in TRPM8 mediated increase in intracellular Ca^2+^ level in 16HBE cells. Intracellular Ca^2+^ levels were measured by Fluo3-AM fluorescent probe assay. **(A)** Cells were exposed to 18°C or/and CSE for 30 min. **(B)** Representative images of fluorescence-positive cells were exposed to18°C for 6 min, to CSE for 3 min, to both cold temperature and CSE for 6 min and with or without transfection of TRPM8 shRNA or scramble shRNA. **(C)** Fluorescence intensity for intracellular calcium concentration was analyzed with the quantification tools. Data in each group are mean ± SD; *n* = 4. ^∗^*p* < 0.05 vs. control, ^#^*p* < 0.05 vs. 18°C alone, ^Δ^*p* < 0.05 vs. CSE alone.

Similar changing tendencies of intracellular calcium were observed in 16HBE cells when they exposed to 18°C and/or CSE. A rapid increase in intracellular Ca^2+^ started at 1 min after treatment initiation, which peaked at 3, 6, or 6 min after exposure to CSE, 18°C or both CSE and 18°C, respectively. In all three groups, it declined somewhat at 10 min after treatment initiation to a plateau that was still higher than the baseline level at the end of the observation period (30 min) (Figure 3A).

Since intracellular Ca^2+^ was highest at 3 min or 6 min after CSE or cold temperature intervention initiation respectively, we chose the time points of 3, 6, and 6 min after 3% CSE, 18°C and CSE, and 18°C intervention, respectively, to compare the effect of 18°C and/or CSE on the elevation of intracellular Ca^2+^ and to explore the impact of inhibition of TRPM8 on the change in intercellular Ca^2+^ induced by 18°C and/or CSE. We found that the co-presence of CSE and 18°C had a stronger effect on the increase in intracellular Ca^2+^ level than 18°C or CSE alone (both *p* < 0.0001, Figure 3B,C). Further analysis revealed that the increase in intracellular Ca^2+^ after 18°C exposure was totally inhibited by transfection of TRPM8 shRNA (*p* < 0.001). However, the increased intracellular Ca^2+^ caused by CSE exposure was not blocked by transfection of TRPM8 shRNA (*p* = 0.213), and the increased Ca^2+^ in 16HBE cells treated with both CSE and 18°C was attenuated by TRPM8 shRNA transfection to a similar level to the CSE-alone group (*p* = 0.322). Inhibition of TRPM8 by pretreatment with BCTC is similar to the TRPM8 shRNA transfection (Supplementary Figure S1). Collectively, these results indicate that both18°C and CSE were capable of increasing intracellular Ca^2+^, but in contrast to the TRPM8-mediated Ca^2+^ influx in 16HBE cells exposed to 18°C, the increase in intracellular Ca^2+^ caused by CSE was TRPM8-independent.

### Roles of CSE in the Cold Temperature-Induced Inflammatory Cytokines Release

**FIGURE 4:**
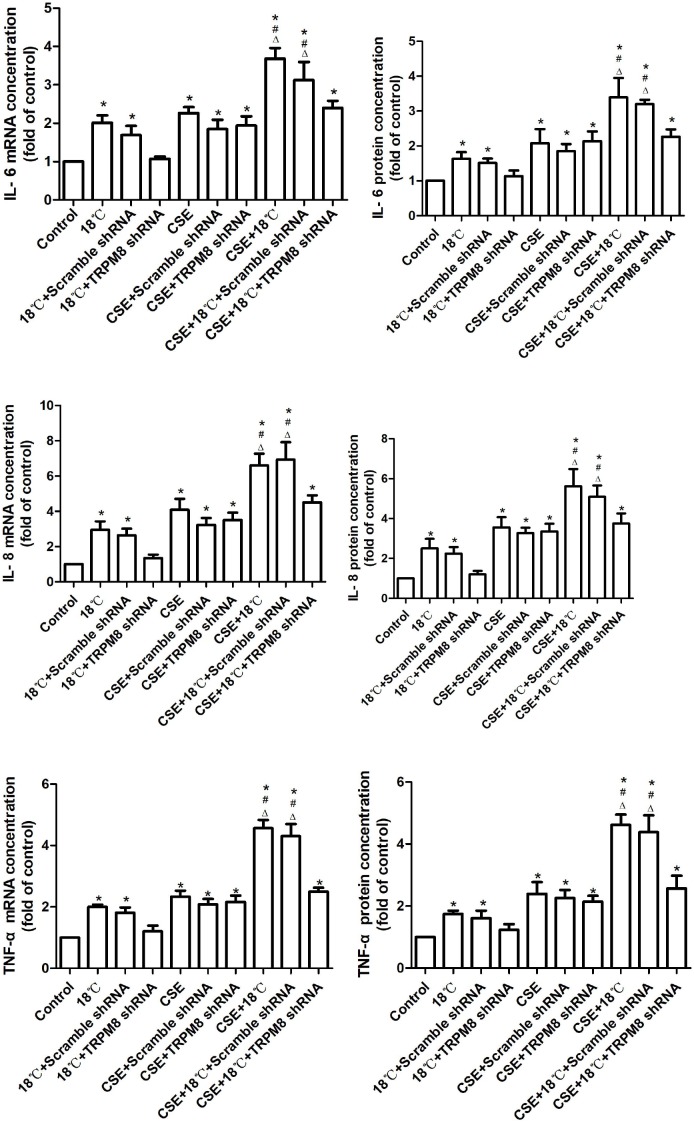
Effects of CSE on cold temperature-induced production of inflammatory cytokines. Quantification of IL-6, IL-8, and TNF-α mRNA and protein in 16HBE cells exposed to 18°C or/and CSE. mRNA and protein expression were evaluated by using real-time PCR with the ΔΔCt method and ELISA assay. Data in each group are mean ± SD; *n* = 4. ^∗^*p* < 0.05 vs. control, ^#^*p* < 0.05 vs. cold alone, ^Δ^*p* < 0.05 vs. CSE alone.

Quantitative real-time PCR and ELISA were used to quantify changes in the mRNAs and proteins of IL-6, IL-8, and TNF-α following exposure of 16HBE cells to CSE and/or 18°C. As shown in Figure 4, both CSE and 18°C alone led to significant increases in the mRNAs and proteins of IL-6, IL-8, and TNF-α following treatment of 16HBE cells (*p* < 0.001). Moreover, the increased IL-6, IL-8, and TNF-α production induced by 18°C was significantly attenuated by transfection of TRPM8 shRNA (*p* < 0.014), whereas the transfection of scramble shRNA did not inhibit the mRNA or protein level of these cytokine production.

To further explore the role of CSE on the 18°C-induced inflammatory cytokine production, we co-treated 16HBE cells with CSE and 18°C and found that such co-exposure induced the mRNAs and proteins of IL-6, IL-8, and TNF-α to a greater extent than stimulation with 18°C or CSE alone [between 1.61- and 2.68-fold increases in the total amounts of IL-6, IL-8, and TNF-α mRNA and protein (all *p* < 0.001)], and the transfection of TRPM8 shRNA effectively reduced the IL-6, IL-8, and TNF-α mRNAs and proteins to levels similar to the CSE exposure group (*p* > 0.5). Inhibition of TRPM8 by pretreatment with BCTC is similar to the TRPM8 shRNA transfection (Supplementary Figure S2).

Overall, these data indicate that CSE can synergistically amplify airway inflammatory cytokine expression induced by cold temperature in a TRPM8-mediated manner.

### Roles of MAPK/NF-κB Signaling Pathway in the Cold Temperature-Induced Production of Inflammatory Cytokines in 16HBE Cells

**FIGURE 5:**
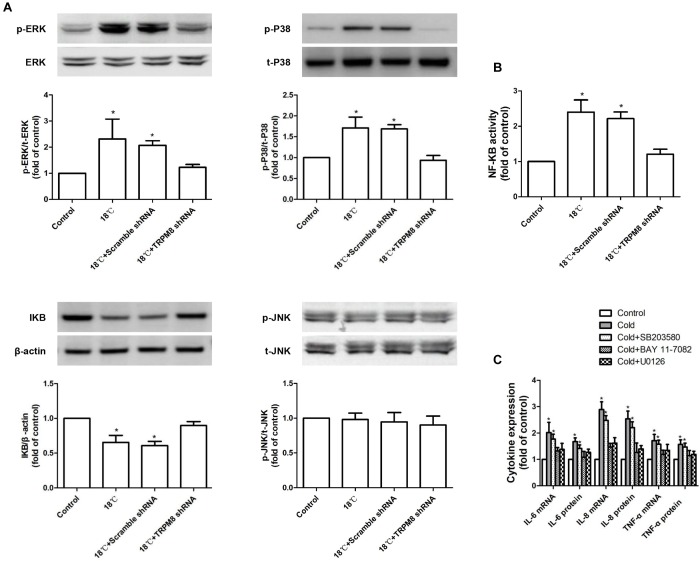
Roles of MAPKs/NF-κB signaling pathway in the cold temperature-induced production of inflammatory cytokines in 16HBE cells. **(A)** Protein expression of ERK, P38, JNK, and IκBα was analyzed by western blotting in 16HBE cells exposed to medium, 18°C, and 18°C with TRPM8 shRNA transfection or scramble shRNA transefection, respectively. **(B)** NF-KB activity was measured with luciferase assay in 16HBE cells exposed to medium, 18°C, and 18°C with TRPM8 shRNA transfection or scramble shRNA transefection, respectively. **(C)** mRNA and protein expression of IL-6, IL-8, and TNF-α were separately measured with RT-RCR and ELISA in 16HBE cells exposed to 18°C with pretreatment with an ERK inhibitor (U0126), a P38 inhibitor (SB203580), or a NF-*κ*B inhibitor (BAY 11-7085; BAY), p- and t- represent phospho- and total-, respectively. Data in each group are mean ± SD *n* = 3. ^∗^*p* < 0.05 vs. control.

To determine whether cold temperature-induced TRPM8-mediated inflammatory cytokine production in 16HBE cells depended on the MAPK/NF-κB signaling pathway, we measured the phosphorylated JNK, p38, and ERK protein expression using western blot analysis and measured the activity of NF-κB using luciferase assay. As shown in Figure 5A,B, exposure to 18°C resulted in obviously increased phosphorylated ERK (control vs. 18°C, 1 ± 0.00 vs. 2.32 ± 0.76, *p* < 0.0001), phosphorylated p38 (control vs. 18°C, 1 ± 0.00 vs. 1.71 ± 0.26, *p* = 0.002), and NF-κB activity (control vs. 18°C, 1 ± 0.00 vs. 2.4 ± 0.42, *p* < 0.001). No difference was observed in the amount of phosphorylated JNK between the 18°C group and control group (control vs. 18°C, 1 ± 0.00 vs. 0.98 ± 0.09, *p* = 0.37). As a key cytoplasmic inhibitor of NF-κB, the degradation of IκBα protein leads to the release and activation of the NF-κB. We also measured the impact of cold temperature on the IκBα protein level and found that compared with the control, exposure of 16HBE cells to 18°C caused a significant reduction of IκBα protein (Figure 5A control vs. 18°C, 1 ± 0.00 vs. 0.65 ± 0.09, *p* = 0.37, *p* = 0.024). Further analysis revealed that such 18°C-induced activation of ERK/p38 signaling and reductions of IκBα protein as well as enhanced NF-κB activity were significantly attenuated by transfection with TRPM8 shRNA (Figure 5A,B, *p* ≤ 0.012), By contrast, following TRPM8 shRNA transfection, the amount of phosphorylated JNK was not significantly different from that in the 18°C stimulation group (*p* = 0.457). Transfection of scramble shRNA transfection did not alter the protein level of ERK or p38, or the activity of NF-κB (*p* ≥ 0.32). Inhibition of TRPM8 by pretreatment with BCTC is similar to the TRPM8 shRNA transfection (Supplementary Figure S3).

The above results indicate that cold temperature could increase the activation of p38 and ERK and simultaneously enhance NF-KB activity by reducing IκBα. To determine whether p38/ERK/NF-κB signaling is necessary for the TRPM8-mediated cold temperature-induced inflammatory cytokine production, we pre-incubated 16HBE cells with a specific inhibitor of ERK (10 μM of U0126), p38 (10 μM SB203580), or NF-κB (10 μM BAY 11-7082) before exposure to 18°C. We found that both U0126 and BAY 11-7082 separately abolished the 18°C-mediated upregulation of IL-6, IL-8, and TNF-α (Figure 5C, *p* < 0.0034), whereas SB203580 exerted a less potent downregulation of this effect (Figure 5C, *p* > 0.076). These results demonstrate that the ERK/NF-κB signaling pathway was primarily involved in TRPM8-mediated cold-induced inflammatory cytokine production.

### Roles of MAPKs/NF-κB Signaling in the Synergistic Effect of CSE on Cold Temperature-Induced Production of Inflammatory Cytokines

**FIGURE 6:**
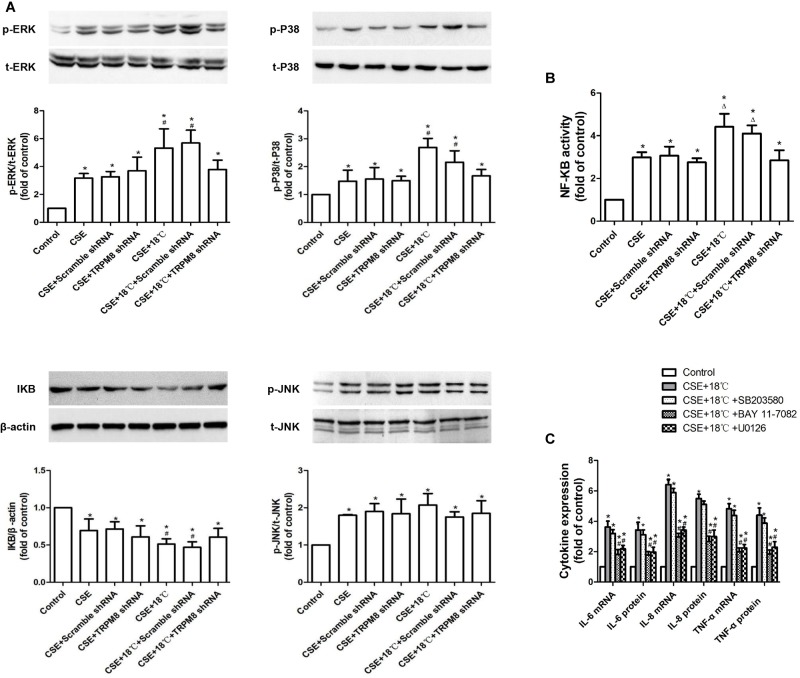
Roles of MAPKs/NF-κB signaling pathway in the synergistic effect of CSE on the cold temperature-induced production of inflammatory cytokines in 16HBE cells. **(A)** Protein expression of ERK, P38, JNK, and IκBα was analyzed by western blotting in 16HBE cells exposed to medium, CSE, both CSE and 18°C with or without transfection of TRPM8 shRNA or scramble shRNA, respectively. **(B)** NF-KB activity was measured with luciferase assay in 16HBE cells exposed to medium, CSE, both CSE and 18°C with or without transfection of TRPM8 shRNA or scramble shRNA, respectively. **(C)** mRNA and protein expression of IL-6, IL-8, and TNF-α were separately measured with RT-RCR and ELISA in 16HBE cells exposed to both CSE and 18°C with pretreatment with an ERK inhibitor (U0126), a P38 inhibitor (SB203580), or a NF-κB inhibitor (BAY 11-7085). p- and t- represent phospho- and total-, respectively. Data in each group are mean ± SD; *n* = 3. ^∗^*p* < 0.05 vs. control, ^#^*p* < 0.05 vs. cold alone, ^Δ^*p* < 0.05 vs. CSE alone.

Next, we checked whether the MAPK/NF-κB signaling pathway participated in the above-observed synergy effect. As shown in Figure 6A,B, the CSE group showed higher levels of phosphorylation of ERK (control vs. CSE, 1 ± 0.00 vs. 3.17 ± 0.47, *p* < 0.001), p38 (control vs. CSE, 1 ± 0.00 vs. 1.57 ± 0.39, *p* = 0.021), and JNK (control vs. CSE, 1 ± 0.00 vs. 1.79 ± 0.19, *p* = 0.017); more enhanced activity of NF-κB (control vs. CSE, 1 ± 0.00 vs. 2.97 ± 0.59, *p* < 0.001); and lower IκBα protein (control vs. CSE, 1 ± 0.00 vs. 0.69 ± 0.11, *p* = 0.016) than the control group. The group co-stimulated with CSE and 18°C showed more markedly increased phosphorylation of ERK (CSE vs. CSE vs.CSE+18°C, 3.17 ± 0.47 vs. 5.19 ± 0.95, *p*<0.0001) and p38 (CSE vs. CSE+18°C, 1.57 ± 0.39, vs. 2.31 ± 0.41 *p* = 0.025) and enhanced activity of NF-κB (CSE vs. CSE+18°C, 2.97 ± 0.5 vs. 4.41 ± 0.91, *p* = 0.017), as well as reduced IκBα protein (CSE vs. CSE vs. CSE+18°C, 0.69 ± 0.11 vs. 0.47 ± 0.073, *p* < 0.001) compared with the CSE group. Furthermore, TRPM8 shRNA transfection significantly attenuated such alterations in protein levels of IκBα, phosphorylated ERK, and phosphorylated p38 as well as the activity of NF-κB (*p* ≤ 0.021). No difference was observed in the amount of phosphorylated JNK between the CSE group and the group co-stimulated with CSE and 18°C (CSE vs. CSE+18°C, 1.79 ± 0.19 vs. 2.01 ± 0.31, *p* = 0.068), and both remained higher than that in the control group (*p* < 0.001). Inhibition of TRPM8 by pretreatment with BCTC is similar to the TRPM8 shRNA transfection (Supplementary Figure S4).

Also, to investigate whether the activation of MAPK/NF-κB signaling participated in the synergistic effect of CSE, specific inhibitor of ERK (10 μM of U0126), p38 (10 μM SB203580), or NF-κB (10 μM BAY 11-7082) were added. Both U0126 and BAY 11-7082 were found attenuate the upregulation of IL-6, IL-8, and TNF-α induced by the co-treatment of CSE and 18°C (Figure 6C, *p* < 0.0017), while the inhibitory effect of SB203580 was not significantly (Figure 5C, *p* > 0.112). These results indicated that the ERK/NF-κB signaling plays a role in the synergistic effect of CSE on cold temperature-induced inflammatory cytokine.

## Discussion

The respiratory tract continuously interacts with the external environment. Cold temperature exposure is a notable environmental factor that elicits a series of effects on the respiratory system, including bronchoconstriction, increased mucous secretions, recruitment of inflammatory cell infiltration, and elevated inflammatory cytokine release (D’Amato et al., 2018). These responses may have some protective roles in healthy individuals when they are exposed to a potentially hazardous cold environment. Unfortunately, they are the most common causes of the acute exacerbation of COPD (Donaldson et al., 1999). Therefore, an in-depth exploration of the molecular and biochemical pathways of cold in lung cells was very important for the prevention of cold-induced acute exacerbation in these diseases.

In this study, we investigated the signaling pathway through which TRPM8 mediated the inflammatory cytokine production in response to cold temperature. We found that exposure of 16HBE cells to 18°C activated the TRPM8 receptor, which sequentially induced an increase in intracellular Ca^2+^, the activation of the ERK/NF-κB signaling, ultimately, the release of IL-6, IL-8, and TNF-α. Additionally, concurrent CSE markedly enhanced the levels of 18°C-induced IL-6, IL-8, and TNF-α production, which resulted from the increased expression of TRPM8 receptor that was followed by a more pronounced intracellular Ca^2+^ elevation and a stronger activation of MAPK/NF-κB signaling. We used this *in vitro* model to elucidate the underlying cellular mechanism of TRPM8-mediated inflammatory cytokine production induced by cold temperature and examined the hypothesis that CSE synergistically enhances this production and the relevant cellular mechanisms.

Chronic obstructive pulmonary disease is a worldwide public health problem that is predicted to be the third leading cause of death globally by 2020 (Murray and Lopez, 1997). Since cigarette smoke has been regarded as the primary contributing factor of COPD (Mannino and Buist, 2007; Ballweg et al., 2014), while cold temperature as a major environmental aggravation stimulus (Donaldson et al., 1999; Tseng et al., 2013), we hypothesized that the co-presence of cigarette smoke and cold temperature would result in a synergistic enhancement of airway inflammation. We demonstrated for the first time that CSE synergistically amplified the production of inflammatory cytokines induced by cold temperature in 16HBE cells. Further, we showed that the observed synergistic production of inflammatory cytokines was based on the upregulation of TRPM8 channel caused by CSE.

As a well-established cold-sensing TRP channel, TRPM8 is a Ca^2+^-permeable channel directly activated by cold temperature. The intracellular Ca^2+^ increase following TRPM8 activation probably consists of two parts. The major portion is the influx of extracellular Ca^2+^ and the Ca^2+^ release from endoplasmic reticulum (ER) through this channel located at the plasma membrane and ER membrane, respectively (Kondratskyi et al., 2013). In addition, calcium-induced activation of phospholipase (PLC) hydrolyzes phosphatidylinositol 4,5-bisphosphate (PIP2), yielding inositol 1,4,5-trisphosphate (IP3), and diacylglycerol (DAG). IP3 can also induce Ca^2+^ release from ER by binding to its calcium channel-coupled receptor on the ER membrane (Bosanac et al., 2004). In the present study, Ca^2+^ imaging revealed a rapid increase in intracellular Ca2^+^ in 16HBE cells exposed to cold temperature (18°C) and/or CSE. However, unlike cells exposed to 18°C, this increase resulting from CSE exposure could not be attenuated with TRPM8 transfection. These results indicate that the CSE-induced intracellular calcium increase was TRPM8 independent. In fact, activation of other TRP channels, such as TRP ankyrin 1 (TRPA1) (Lin et al., 2015), TRP canonical 1 (TRPC1), and TRPC6 (Wang et al., 2014), have been reported to lead to an increase in Ca^2+^ influx caused by cigarette smoke. Further studies are needed to determine whether CSE-induced Ca^2+^ influx is TRPC or TRPA dependent or is it due to excessive Ca^2+^ load.

Abnormal inflammatory response in the lung and airway is regarded as a key mechanism in the pathogenesis of COPD. Multiple inflammatory mediators derived from inflammatory cells and structural cells of the airway and lung are increased in COPD. Once provoked, epithelial cells produce inflammatory mediators, such as IL-1, IL-6, IL-8, TNF-α, and GM-CSF (Angelis et al., 2014; Barnes, 2016). In the present study, we also showed that cold temperature or CSE alone was able to upregulate the production of IL-6, IL-8, and TNF-α in 16HBE cells. More importantly, we demonstrated that CSE synergistically amplified these cytokines induced by cold temperature. The proinflammatory cytokines IL-6 and TNF-α are responsible for the initiation and amplification of inflammatory reactions. In addition, TNF-α activated NF-κB, a master switch for many genes, including IL-8 and MUC5AC. IL-8 is a potent chemoattractant and activator for neutrophils and monocytes, which are major sources of matrix metalloproteinase-9 and neutrophil elastase, proteins that are central to the processes of emphysema and mucus hypersecretion (Barnes, 2009). Our present study showed that CSE-induced amplification of IL-6, IL-8, and TNF-α was effectively blocked by the transfection of TRPM8 shRNA, demonstrating the essential role of TRPM8 for this synergy to occur.

Mitogen-activated protein kinase and NF-κB are the most activated intracellular signaling molecules in airway epithelial cells associated with inflammatory cytokine and chemokine release (Kim and Choi, 2010; Yan and Choi, 2014). MAPKs, a large family of serine/threonine kinases, are central inflammatory signaling pathways from the cell surface to the nucleus (Roux and Blenis, 2004), while NF-κB is a nuclear transcription factor that is typically inactived within the cytoplasm due to its interaction with IκB proteins that mask its nuclear localization sequence (NLS) under normal condition (Oeckinghaus and Ghosh, 2009). Activation of MAPKs and NF-κB by co-presence of stress and proinflammatory cytokines has long been known and is often attributed to common components, but the relationship between the activation of the MAPKs and NF-κB remains controversial. Some studies suggest that NF-κB is a downstream component of the MAPK signaling pathway (Sun et al., 2013), but another study demonstrated that activation of p38 and NF-κB was mediated by separate pathways, which may converge further downstream, in the cell nucleus (Wesselborg et al., 1997). Our study indicated an association between the synergistic induction of inflammatory cytokines by CSE and the activity changes in MAPK and NF-κB signaling, but it does not conclusively demonstrate whether MAPK and NF-κB are upstream or downstream of a certain signaling pathway.

## Author Contributions

ML and XZ are the guarantors and designed the research. JW and GY performed the research and analyzed the data. JW wrote the manuscript.

## Conflict of Interest Statement

The authors declare that the research was conducted in the absence of any commercial or financial relationships that could be construed as a potential conflict of interest.
