# Supplementary material for: Transient Receptor Potential Melastatin 8 (TRPM8)-Based Mechanisms Underlie Both the Cold Temperature-Induced Inflammatory Reactions and the Synergistic Effect of Cigarette Smoke in Human Bronchial Epithelial (16HBE) Cells
Source: Front Physiol. 2019 Mar 29;10:285. doi: 10.3389/fphys.2019.00285 (PMC6455074; doi:10.3389/fphys.2019.00285)
Supplement: Supplementary file 1 [file Data_Sheet_1.PDF]

(A)

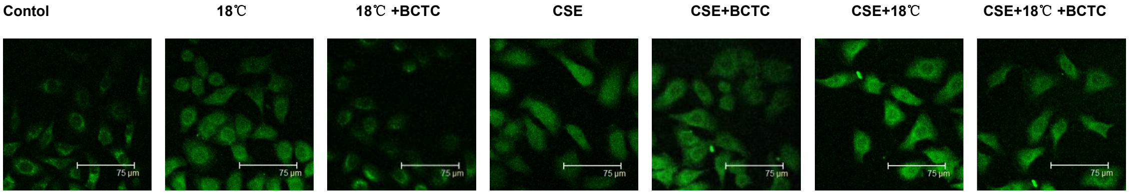

(B)

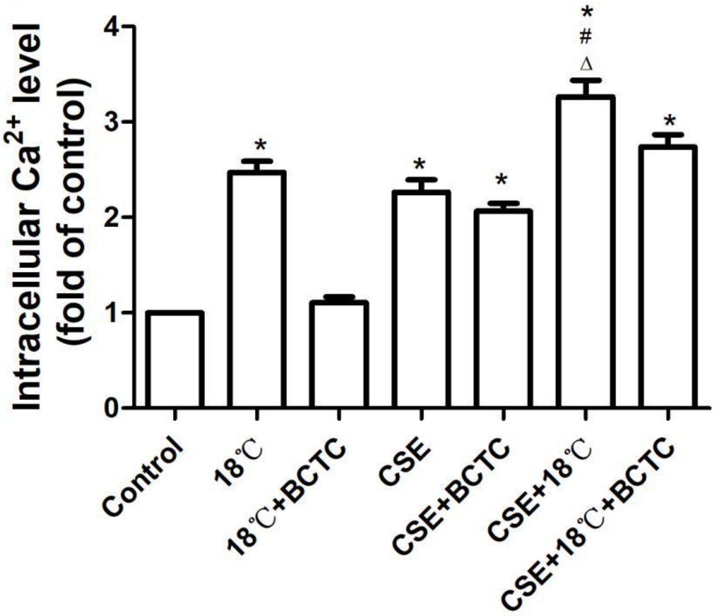

Figure S1. Roles of cold temperature or/and CSE in TRPM8 mediated increase in intracellular Ca^{2+} level in 16HBE cells. Intracellular Ca^{2+} levels were measured by Fluo3-AM fluorescent probe assay. (A) Representative images of fluorescence-positive cells were exposed to18°C for 6min, to CSE for 3min, to both cold temperature and CSE for 6min and with or without BCTC pretreatment. (B)Fluorescence intensity for intracellular calcium concentration was analyzed with the quantification tools. Data in each group are mean ± SD; n=4. $^{*}p < 0.05$ versus control, $^{\#}p < 0.05$ versus 18°C alone, $^{\Delta}p < 0.05$ versus CSE alone.

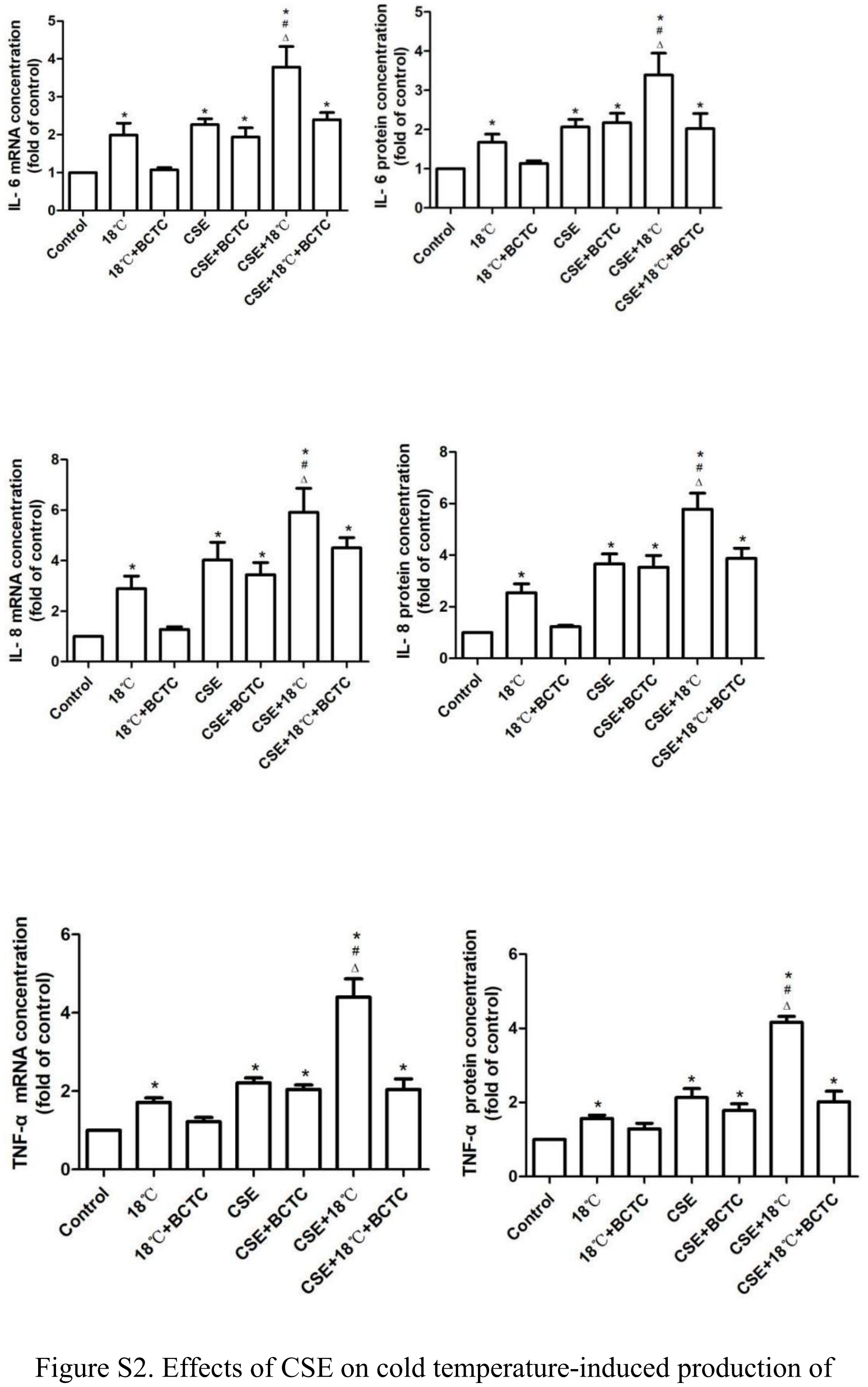

Figure S2. Effects of CSE on cold temperature-induced production of inflammatory cytokines. Quantification of IL-6, IL-8 and TNF-α mRNA and protein in 16HBE cells exposed to 18℃ or/and CSE. mRNA and protein expression were evaluated by using real-time PCR with the ΔΔCt method and ELISA assay. Data in each group are mean ± SD; n=4. $^{*}p < 0.05$ versus control, $^{\#}p < 0.05$ versus cold alone, $^{\Delta}p < 0.05$ versus CSE alone.

（A）

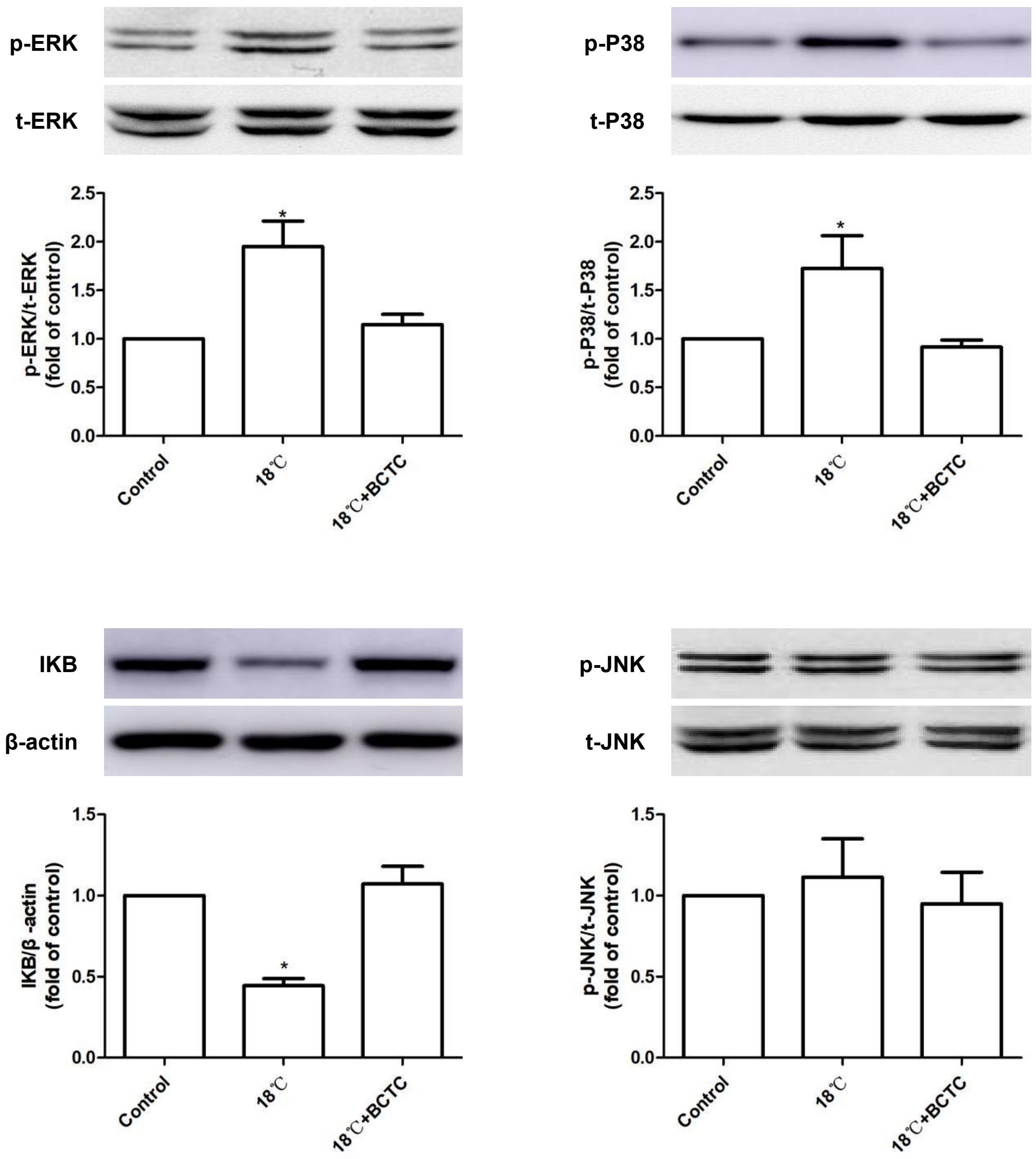
p-ERK
t-ERK
p-P38
t-P38
p-ERK/t-ERK
(fold of control)
p-P38/t-P38
(fold of control)
2.5
2.0
1.5
1.0
0.5
0.0
*
Control
18℃
18℃+BCTC
IKB
β-actin
p-JNK
t-JNK
IKB/β -actin
(fold of control)
p-JNK/t-JNK
(fold of control)
1.5
1.0
0.5
0.0

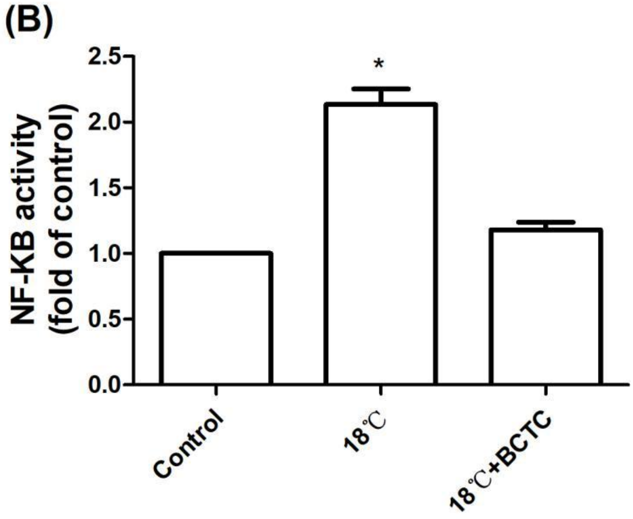

Figure S3. Roles of MAPKs/NF-κB signaling pathway in the cold temperature-induced production of inflammatory cytokines in 16HBE cells. (A) Protein expression of ERK, P38, JNK and IκBα was analyzed by Western blotting in 16HBE cells exposed to medium, 18°C and 18°C with BCTC pretreatment. (B) NF-KB activity was measured with luciferase assay in 16HBE cells exposed to medium, 18°C and 18°C with BCTC pretreatment. p- and t- represent phospho-, total- respectively. Data in each group are mean ± SD; n=3. *$p < 0.05$ versus control.

(A)

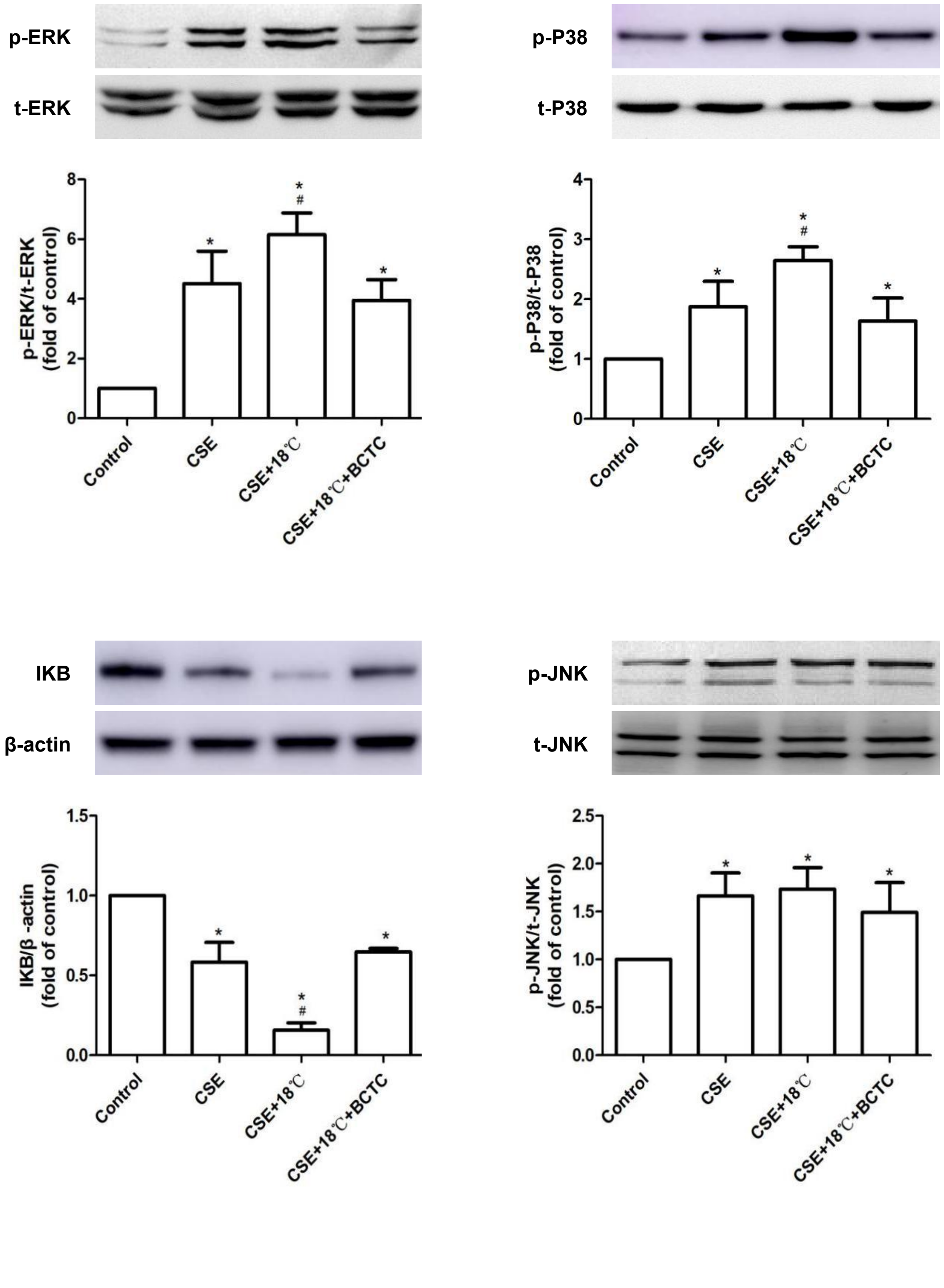

p-ERK
t-ERK
p-P38
t-P38
p-ERK/t-ERK (fold of control)
p-P38/t-P38 (fold of control)
Control
CSE
CSE+18℃
CSE+18℃+BCTC
IKB
β-actin
p-JNK
t-JNK
IKB/β -actin (fold of control)
p-JNK/t-JNK (fold of control)

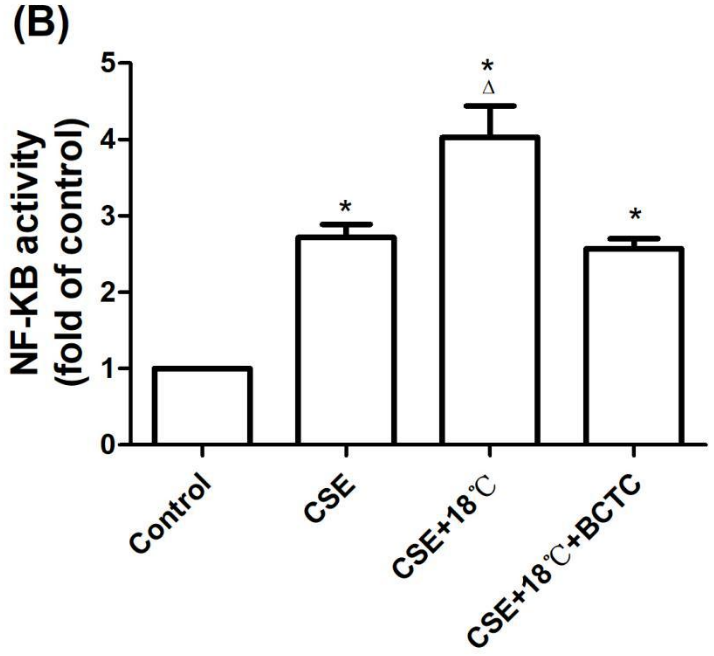

Figure S4. Roles of MAPKs/NF-κB signaling pathway in the synergistic effect of CSE on the cold temperature-induced production of inflammatory cytokines in 16HBE cells. (A) Protein expression of ERK, P38, JNK and IκBα was analyzed by Western blotting in 16HBE cells exposed to medium, CSE, both CSE and 18°C with or without BCTC pretreatment. (B) NF-KB activity was measured with luciferase assay in 16HBE cells exposed to medium, CSE, both CSE and 18°C with or without BCTC pretreatment. p- and t- represent phospho-, total- respectively. Data in each group are mean ± SD; n=3. $^*p < 0.05$ versus control. $^{\Delta}p < 0.05$ versus CSE alone.
